# Dysregulation of Labile Iron Predisposes Chemotherapy Resistant Cancer Cells to Ferroptosis

**DOI:** 10.3390/ijms26094193

**Published:** 2025-04-28

**Authors:** Luke V. Loftus, Louis T. A. Rolle, Bowen Wang, Kenneth J. Pienta, Sarah R. Amend

**Affiliations:** 1 Cellular and Molecular Medicine Program, Johns Hopkins School of Medicine, Baltimore, MD 21287, USA; 2 Cancer Ecology Center, Brady Urological Institute, Johns Hopkins School of Medicine, Baltimore, MD 21287, USA; 3 Pathobiology Program, Johns Hopkins School of Medicine, Baltimore, MD 21287, USA

**Keywords:** ferroptosis, labile iron, chemotherapy, therapeutic resistance

## Abstract

Despite centuries of research, metastatic cancer remains incurable due to resistance to all conventional cancer therapeutics. Alternative strategies leveraging non-proliferative vulnerabilities in cancer are required to overcome cancer recurrence. Ferroptosis is an iron dependent cell death pathway that has shown promising pre-clinical activity in several contexts of therapeutic resistant cancer. However, ferroptosis sensitivity is highly variable across tissue types and cell states, posing a challenge for clinical translation. We describe a convergent phenotype induced by chemotherapy where cells surviving chemotherapy have dysregulated iron homeostasis, regardless of initial cell type or chemotherapy used. Elevated labile iron levels are counteracted by NRF2 signaling, yet the resulting antioxidant programs do not alleviate the labile iron burden. Selectively inhibiting GPX4 leads to uniform susceptibility to ferroptosis in surviving cells, highlighting the common reliance on lipid peroxidation defenses. Cellular iron dysregulation is a vulnerability of chemoresistant cancer cells that can be leveraged by triggering ferroptosis.

## 1. Introduction

Once cancer has metastasized, therapeutic resistance is the primary factor preventing durable cancer clearance. Nearly all current therapeutic strategies leverage the high proliferative rate of cancer cells to elicit apoptosis triggered by engagement of cell cycle checkpoints [1]. While anti-cancer therapies initially control tumor burden by eliminating the highly proliferative cancer cells, inevitably, a population of cancer cells evades apoptosis to survive, resulting in tumor recurrence. Non-apoptotic cell death pathways are cell cycle agnostic and are beginning to be explored to overcome therapeutic resistance in cancer.

Ferroptosis was officially named in 2012 as an iron-dependent form of cell death defined by unconstrained lipid peroxidation culminating in physical breakdown of plasma membrane integrity [2,3]. Iron reactivity is central to ferroptosis as iron both initiates lipid peroxidation and is required for amplification of lipid peroxidation through generation of alkoxyl radicals [4]. Glutathione peroxidase 4 (GPX4), the primary cellular defense against lipid peroxidation, prevents lipid hydroperoxide accumulation by converting to lipid alcohols [3]. Ferroptosis has gained considerable interest as an anti-cancer approach in a variety of drug-tolerant cancer subpopulations, all of which are heavily reliant on lipid peroxidation defenses [5,6,7,8]. Due to its metabolic underpinnings, however, mechanisms underlying ferroptosis sensitivity are highly variable across anatomical location, genetic background, and cellular phenotype over time [9,10,11].

Iron utilization is as evolutionarily ancient as life itself. Planetary iron composition at the time of Earth’s formation was likely deterministic in the origin of biologic chemistry, and iron utilization is evident in the earliest unicellular lifeforms [12,13,14,15]. Subsequent global changes in iron bioavailability shaped selective pressures for iron acquisition, detoxification, and utilization, which spurred evolution of symbiotic relationships and, ultimately, complex multicellular life on Earth [15]. The fact that iron has retained its essential role throughout geological time and in nearly all species (save for two bacterial life forms [16]) highlights its irreplaceable role in numerous vital functions, including DNA replication, transcription, redox sensing, and mitochondrial metabolism [17,18,19]. However, iron’s unique electron transfer properties that enable life also indiscriminately generate toxic radicals when iron is freely reactive [20,21]. Thus, throughout the biochemical and geochemical coevolution of iron utilization, cellular programs to manage iron’s access to oxygen and oxidation state are critical to avoid toxicity.

In humans, cellular iron is imported primarily through endocytosis of the transferrin receptor and liberation of iron in mature lysosomes. Minimal amounts of ferrous iron exist in a dynamic and exchangeable labile iron pool (LIP) before incorporation into cofactors as well as directly into protein active sites [18]. Even in the LIP, iron is predominantly bound to glutathione to minimize spurious reactivity [22,23]. When free iron accumulates intracellularly, it is detoxified and stored as Fe^3+^ in ferritin proteins that evolved along the ancient history of iron utilization [24]. Poly(rC)-binding protein 1 (PCBP1) is the primary iron chaperone shown to directly bind and deliver Fe-GSH to ferritin and several apoenzymes [23,25,26].

Cancer has long been recognized as iron addicted to support proliferation and increased biomass. Less is known about the intracellular management of iron during cancer progression, as well as how cells handle this vital, yet potentially toxic, resource when reacting to therapeutic stress. We have previously shown that cells surviving single dose chemotherapy uncouple DNA replication and cell division leading to increased ploidy and cell size, and the surviving cells are resistant to subsequent chemotherapy [27]. To identify convergent phenotypes and shared vulnerabilities of chemotherapy resistance, we evaluated two cell lines of different tumor types (PC3; metastatic prostate cancer cell line and MDA-MB-231; metastatic breast cancer cell line) and two classes of chemotherapy (DNA damaging cisplatin and microtubule stabilizer docetaxel). We found that cells surviving chemotherapy have high amounts of labile iron regardless of cell line or chemotherapy treatment. We identified canonical cellular responses to iron toxicity, including NRF2 signaling, upregulation of glutathione (GSH) biosynthesis, and ferritin expression in all settings. Despite the physiological response to iron accumulation in surviving cells, labile iron was not attenuated, likely due to a relative loss of the PCBP1 chaperone. Labile iron burden in resistant cells conferred uniform sensitivity to ferroptosis via GPX4 inhibition, regardless of originating cell line or chemotherapy. This study highlights labile iron accumulation as a driver of ferroptosis sensitivity in chemoresistant cells, despite active NRF2 signaling. For ferroptosis to translate into a viable clinical option in metastatic cancer settings, the timing of intervention will be critical to maximize efficacy and enable the treatment of multiple tumor types.

## 2. Results

### 2.1. Cells Surviving Chemotherapy Have High Labile Iron

**Figure 1:**
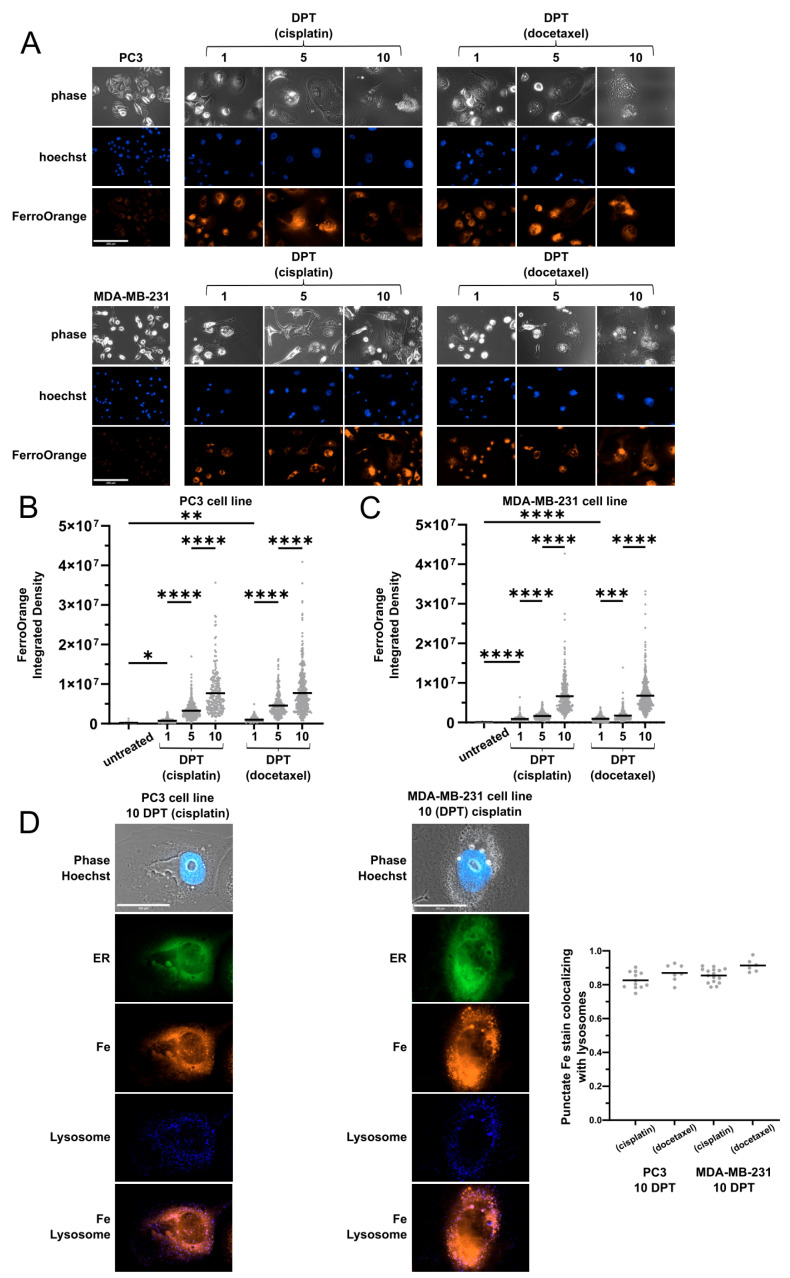
Cells surviving chemotherapy have high amounts of labile iron. (**A**) Live cell images of PC3 and MDA-MB-231 cells before and after cisplatin or docetaxel using FerroOrange. Scale bar: 100 μm. (**B**,**C**) Quantification of FerroOrange integrated density from (**A**). in PC3 (**B**) or MDA-MB-231 (**C**) cells before and after chemotherapy. Data display individual cells (dot) and mean (bar) from three biological replicates. Adjusted *p*-values reported as <0.05 (*), <0.01 (**), <0.001 (***), <0.0001 (****). (**D**) Live cell images with FerroOrange (Fe), ERTracker Green (ER), and LysoTracker (Lysosome) of PC3 and MDA-MB-231 cells 10 days after cisplatin treatment. Scale bar: 100 μm. Quantification of punctate FerroOrange staining colocalizing with LysoTracker staining (right). Data are Manders Colocalization Coefficient for fraction of punctate iron stain colocalized with lysosome.

PC3 prostate and MDA-MB-231 breast cancer cell lines were treated with cisplatin or docetaxel for 72 h; then drug was removed, and cells were followed up to 10 days post-treatment (DPT). Consistent with previous reports [27,28], surviving cells increased in size with time (Supplementary Figure S1). Increasing cellular biomass requires heightened metabolism and anabolic precursors, including trace metals such as iron. Physiological regulation of iron maintains low levels of labile iron to accommodate protein synthesis demands but avoid spurious reactivity and oxidative damage [18]. We assessed labile iron by live cell imaging (Figure 1) and an orthogonal lysate-based assay (Supplementary Figure S2B). Labile iron levels were higher in untreated PC3 than MDA-MB-231 cells and were modestly elevated at 1-DPT in both cell lines, regardless of chemotherapy treatment, consistent with de-compartmentalization of iron following acute stress [20] (Figure 1A–C, Supplementary Figure S2A,B). Labile iron increased dramatically from 5 to 10 DPT, in both cell lines and chemotherapy groups (Figure 1A–C, Supplementary Figure S2B), indicating a common mismanagement of cellular iron. Labile iron accumulates primarily in mitochondria and ER due to iron-containing proteins and protein assembly in both compartments [17]. At 10 DPT, labile iron showed visible punctate staining that exceeded ER boundaries, regardless of cell line or chemotherapy, further implying a dysregulation in iron homeostasis. (Figure 1D, Supplementary Figure S2C). Punctate labile iron staining in cells 10 DPT showed large overlaps with active lysosomes (Figure 1D, Supplementary Figure S2C).

### 2.2. Ferritin Expression Does Not Attenuate Labile Iron Levels

**Figure 2:**
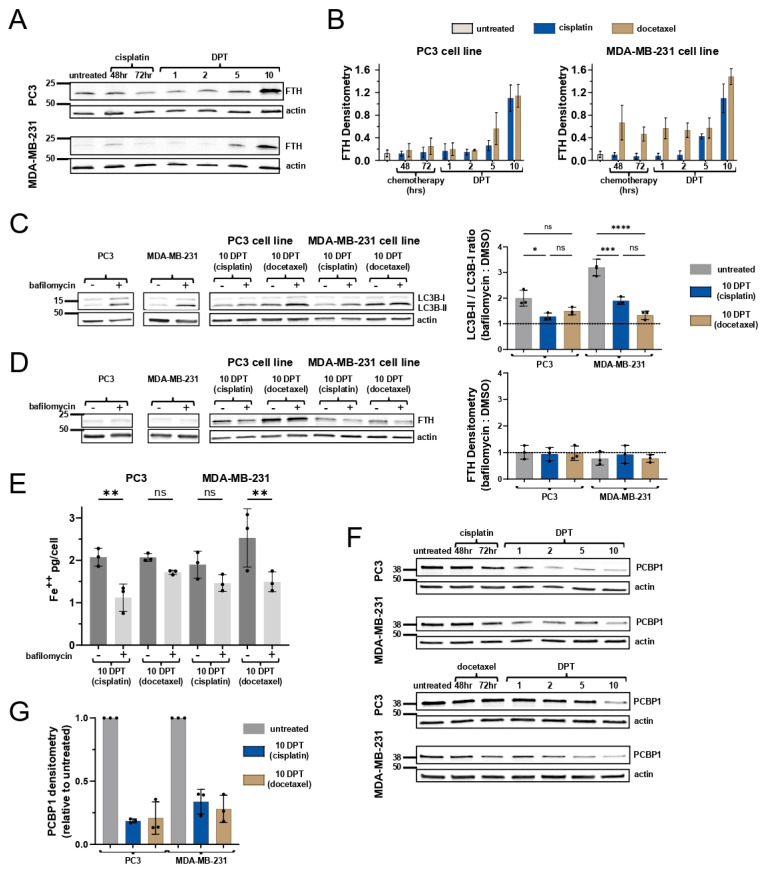
Cells surviving chemotherapy have high ferritin expression that is not being turned over by autophagy. (**A**) Western blot for ferritin in PC3 and MDA-MB-231 cells before and after cisplatin. (**B**) Quantification of ferritin expression from A. Data are mean and standard deviation of three biological replicates. (**C**) Western blot for LC3B expression with and without bafilomycin treatment in PC3 and MDA-MB-231 cells surviving chemotherapy. Quantification of LC3B-II/LC3B-I ratio in bafilomycin/DMSO condition (right). Dotted line at 1 represents no change from DMSO-treated LC3B ratio. (**D**) Western blot for ferritin expression with and without bafilomycin treatment in PC3 and MDA-MB-231 cells surviving chemotherapy. Quantification of ferritin in bafilomycin/DMSO condition (right). Dotted line at 1 represents no change from DMSO-treated ferritin expression. (**E**) Labile iron quantification with and without bafilomycin treatment in PC3 and MDA-MB-231 cells surviving chemotherapy. Adjusted *p*-values reported as not significant (ns), <0.05 (*), <0.01 (**), <0.001 (***), <0.0001 (****). (**F**) Western blot for PCBP1 in PC3 and MDA-MB-231 cells surviving chemotherapy. (**G**) Quantification of PCBP1 expression from G. in 10 DPT cells relative to respective untreated cells.

In response to labile iron accumulation, cells detoxify and store Fe^3+^ in ferritin [29]. Untreated cells lines and early DPT do not have appreciable ferritin expression, but by 10 DPT there is a marked increase in ferritin across all samples (Figure 2A,B, Supplementary Figure S2D). Paradoxically, this elevated ferritin expression does not accompany a decrease in the LIP in cells 10 DPT (Figure 1A–C, Supplementary Figure S2B). In addition, transferrin receptor protein expression is unchanged (Supplementary Figure S2E), suggesting a disconnect between surviving cells’ requirement for iron utilization vs. storage.

To further investigate iron homeostasis, we next evaluated autophagy as selective ferritin degradation, ferritinophagy, is a well characterized contributor to labile iron accumulation [3]. Cells 10 DPT have an elevated LC3B-II-to-LC3B-I ratio indicative of increased autophagosome formation (Supplementary Figure S3A). However, upon the inhibition of autophagy with bafilomycin, there is less accumulation of LC3B at 10 DPT than in untreated cells, indicating decreased autophagic flux in surviving cells (Figure 2C). Further, bafilomycin treatment does not lead to the accumulation of ferritin, meaning ferritin is not being degraded through autophagy in all cells pre- and post-treatment with chemotherapy (Figure 2D). Accordingly, autophagy is not contributing to the elevated labile iron levels in surviving cells (Figure 2E). Bafilomycin interferes with lysosomal biology, not only autolysosomes, which, alongside the lysosomal accumulation of iron, likely explains partial labile iron modulation by bafilomycin in 10 DPT surviving cells (Figure 2E).

In the absence of ferritinophagy, we evaluated iron trafficking as a mechanism underlying increased iron in surviving cells. PCBP1 is the primary iron chaperone known to deliver Fe-GSH complexes to ferritin [25]. PCBP1 is required for iron loading into ferritin and the loss of PCBP1 causes poor ferritin iron loading, increased labile iron pool, and lipid peroxidation [25,30,31]. PCBP1 is ubiquitously expressed at high levels in mammalian cells, as it is necessary for iron chaperone activity [23], and PCBP1 protein is indeed abundant in untreated cancer cell lines (Figure 2F). However, PCBP1 expression waned over time following cisplatin or docetaxel treatment, with cells 10 DPT being significantly depleted of PCBP1 across all contexts (Figure 2F,G). PCBP1 is not being turned over by autophagy (Supplementary Figure S3B).

Taken together, these data suggest that while cells surviving chemotherapy are responding to labile iron accumulation, they are not effectively sequestering iron, likely due to a relative loss in PCBP1 chaperone.

### 2.3. Surviving Cells Have Increased NRF2-Mediated Antioxidant Response

**Figure 3:**
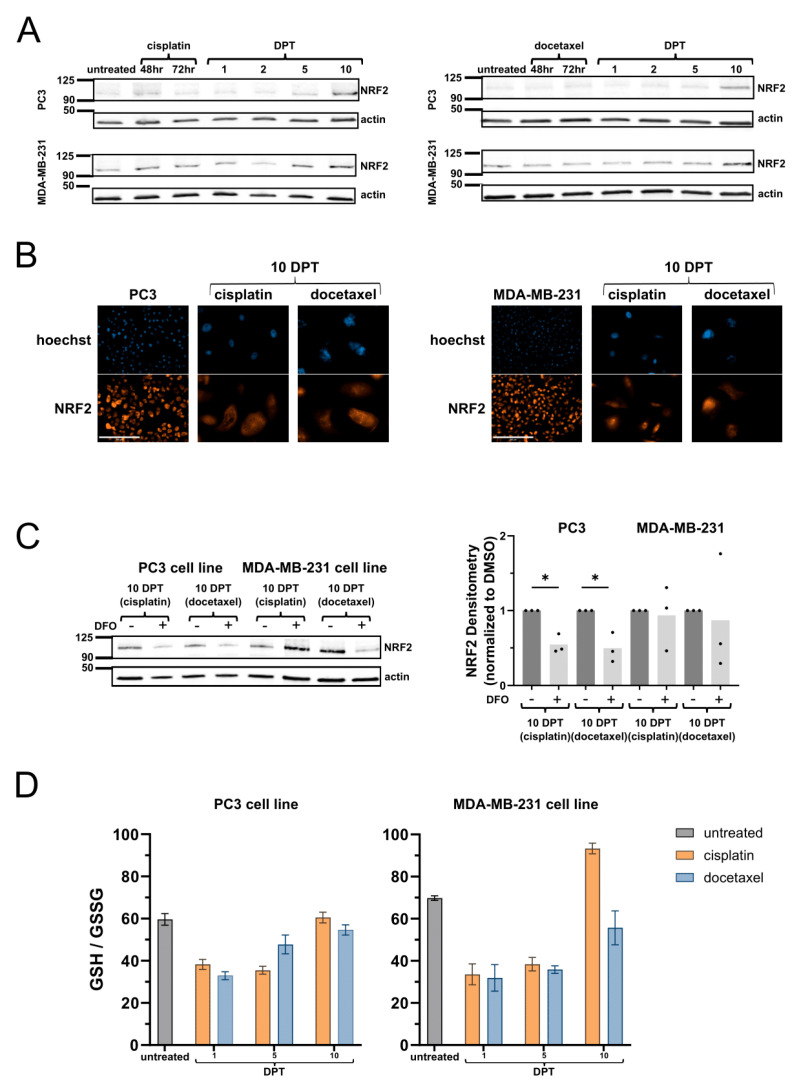
Ten DPT surviving cells have high NRF2 expression and restored GSH ratio. (**A**) Western blot for NRF2 in PC3 and MDA-MB-231 cells before and after cisplatin. (**B**) NRF2 immunofluorescence in PC3 and MDA-MB-231 cells before and after cisplatin or docetaxel. Scale bar: 100 μm. (**C**) Western blot for NRF2 following 6 h of deferoxamine mesylate (DFO) treatment in cells 10 days post-chemotherapy. Adjusted *p*-values reported as <0.05 (*). (**D**) GSH: GSSG ratio in PC3 and MDA-MB-231 cells following cisplatin or docetaxel treatment.

Given excessive labile iron in cells that survive chemotherapy, we next investigated cellular mechanisms that mitigate iron toxicity. Transcription factor NRF2 is a master coordinator of the cellular antioxidant response to oxidative damage, including from heavy metal toxicities [32,33]. MDA-MB-231 cells have slightly higher NRF2 expression than PC3 cells in untreated samples, as well as during chemotherapy and 1 DPT timepoints (Figure 3A). By 10 DPT, surviving cells have high NRF2 expression with prominent nuclear localization, regardless of cell line or chemotherapy (Figure 3A,B). Deferoxamine (DFO) is an iron chelator that attenuates cellular iron import through endocytosis and the chelation of iron released from transferrin in nascent lysosomes [3]. DFO treatment for six hours partly attenuated NRF2 expression in PC3 cells 10 DPT, but not MDA-MB-231 cells 10 DPT, indicating potential differences in active iron import contributing to NRF2 activity (Figure 3C). NRF2 coordinates the expression of thousands of genes, most prominently ferritin (iron sequestration and storage), GSH biosynthesis enzymes (GSH synthesis), and p62 (coordinating protein clearance), which are enriched in genetic or pharmacologic manipulation of NRF2 [34]. Ferritin and p62 are both highly expressed in cells 10 DPT (Figure 2A,B, Supplementary Figures S2D and S4A), suggesting a connection between oxidative stress, inefficient autophagy, and protein clearance.

The ratio of reduced to oxidized glutathione (GSH) is a widely used indicator of oxidative stress. We measured GSH ratio by plate-based assay and found that cells 1 and 5 DPT have a compromised GSH ratio, indicative of general oxidative stress from chemotherapy. By 10 DPT, the GSH ratio is restored to similar levels as untreated cells (Figure 3D), consistent with NRF2-driven enrichment of GSH biosynthesis enzymes. Reduced GSH is the most abundant antioxidant in cells with well-known roles in direct detoxification and the regeneration of antioxidant enzyme active sites. GSH is also the primary ligand for ferrous labile iron, serving as a critical non-chelating ligand that enables intracellular iron trafficking and selectivity over manganese for enzyme incorporation [22,35,36]. It is hypothesized that GSH’s primary role may be iron handling, secondary to its function as a redox buffer, with its high cytosolic abundance preserving its iron handling functions even during fluctuations caused by redox stressors [37]. Based on these results, we hypothesize that in surviving cells at 10 DPT, reduced GSH is both occupied by the excessive amount of labile iron, particularly due to the relative loss of PCBP1, and serving its traditionally described antioxidant roles to counteract oxidative damage from accrued DNA damage and iron-catalyzed reactions.

### 2.4. Cells Surviving Chemotherapy Are Vulnerable to Ferroptosis

**Figure 4:**
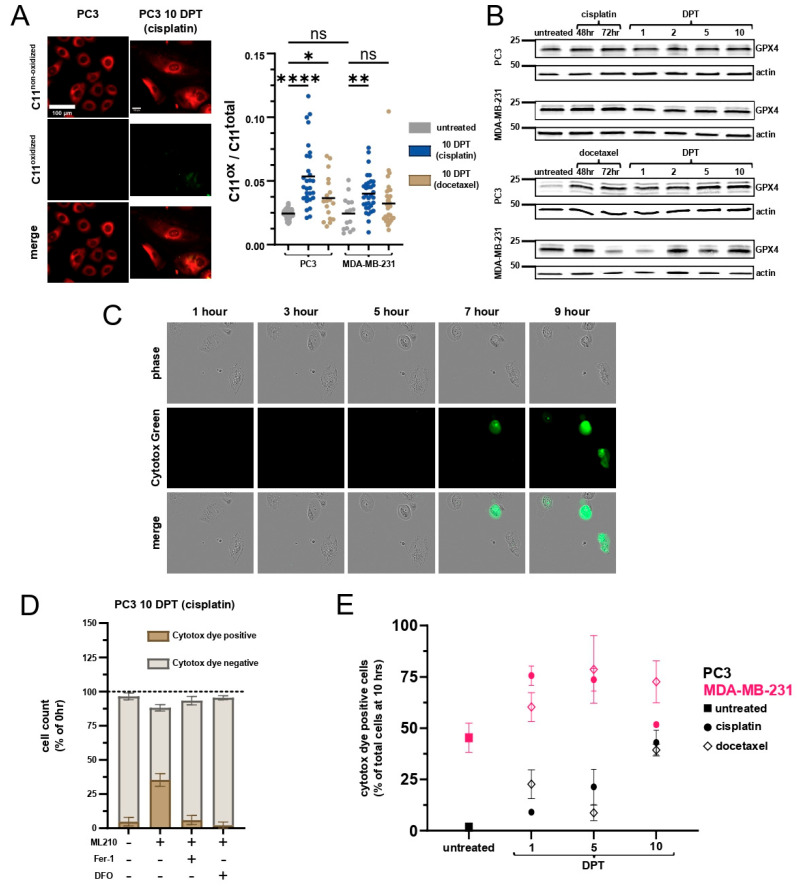
Surviving cells 10 days post-chemotherapy are susceptible to ferroptosis regardless of cell line of origin. (**A**) C11 BODIPY imaging in PC3 cells untreated and 10 days post-cisplatin. Scale bar: 100 μm. Adjusted *p*-values reported as not significant (ns), <0.05 (*), <0.01 (**), <0.0001 (****). Quantification is ratio of oxidized (green) to total (green + red) C11 fluorescence. (**B**) GPX4 expression in PC3 or MDA-MB-231 cells surviving cisplatin or docetaxel. (**C**) Timelapse images of PC3 cells 10 days post-cisplatin treated with ML210. Cytotox dye incorporation indicates membrane permeability. (**D**) Quantification of live cell imaging for PC3 cells 10 days post-cisplatin treated with, from left to right, DMSO (0.1%); ML210 (1 µM); ML210 and Fer-1 (1 µM); and ML210 and DFO (50 µM). Percent of total cell count shows change in cell number over 10 h from cell detachment. CytotoxGreen-positive cells are still adherent at end of timelapse (10 h). Fer-1 = Ferrostatin-1. DFO = deferoxamine. (**E**) Percent of Cytotox dye-positive cells following ML210 (1 µM) treatment across all samples.

Labile iron causes lipid peroxidation by directly reacting with lipid peroxides through Fenton reactions, generating free radicals that damage lipids, and by catalyzing enzyme-dependent lipid peroxide formation [38]. Given the high levels of labile iron and reduced GSH pools in cells surviving chemotherapy, we hypothesized that cell survival is reliant on lipid peroxidation defense systems. We assessed lipid peroxidation using C11 BODIPY reagent and found increased lipid peroxidation in cells at 10 DPT (Figure 4A). Thus, despite the accumulated labile iron, cells at 10 DPT are effectively counteracting lipid peroxidation prior to any intervention.

GPX4 is the only mammalian antioxidant enzyme capable of cleansing lipid peroxidation. GPX4 expression is largely unchanged across all cell lines and timepoints (Figure 4B, Supplementary Figure S5A), suggesting lipid peroxidation is combatted through regeneration, rather than increased expression, of GPX4. We utilized the GPX4 inhibitor ML210 to assess ferroptosis vulnerability. ML210 is a masked nitrile-oxide electrophile that is converted intracellularly to a transient active compound, which then covalently modifies the GPX4 active site [39,40]. Cells were treated with 1 µM dose of ML210 and ferroptosis was quantified over a 10 h period. As expected, GPX4 inhibition causes an increase in lipid hydroperoxide abundance by 6 h (Supplementary Figure S5B). Ferroptosis was quantified by time lapse imaging to account for cell loss from apoptosis (especially in early recovery timepoints following chemotherapy) and visualize the loss of membrane integrity. Susceptible cells undergo membrane blowouts and full loss of membrane integrity, as detected by uptake of a cell impermeable dye, and remain adherent at the 10 h timepoint (Figure 4C, Supplementary Figure S5C, Supplementary Movies S1–S4). To confirm ferroptosis as the mechanism of cell death, cell death was fully prevented in the presence of Ferrostatin-1 (lipid radical scavenger) [41] or deferoxamine (iron chelator) in all samples (Figure 4D, Supplementary Figure S6, Supplementary Movies S5 and S6).

Consistent with the known ferroptosis sensitivity of the MDA-MB-231 cell line [42,43], we find 45% of untreated MDA-MB-231 cells die due to ferroptosis with GPX4 inhibition (Figure 4E). Chemotherapy increased sensitivity to ferroptosis with a peak sensitivity at 5 DPT, where around 75% of MDA-MB-231 cells undergo ferroptosis regardless of chemotherapy group. At 10 DPT, cisplatin-treated cells were less vulnerable to ferroptosis, at levels similar to those of untreated MDA-MB-231 cells. Docetaxel-treated cells retained much of their susceptibility, likely due to the (relatively) lower GSH ratio and marked transcriptional differences from cisplatin-treated cells at 10 DPT (Figure 2A and Figure 4E).

Untreated PC3 cells are agnostic to GPX4 inhibition and cisplatin only modestly increased sensitivity at 1 and 5 DPT. A total of 23% of PC3 cells 1 day post-docetaxel died to ferroptosis, and by 5 days post-docetaxel, sensitivity was only 8%. PC3 cells at 5 DPT docetaxel were not significantly more sensitive to ferroptosis than untreated PC3 cells, likely due to ferritin, GSH pathway, and GSH ratio enrichment at 5 DPT in this context (Figure 4E). By 10 DPT, regardless of chemotherapy, surviving cells were more vulnerable to ferroptosis at similar levels to MDA-MB-231 cells 10 days post-cisplatin (Figure 4E).

Similar vulnerability to ferroptosis at 10 DPT in both cell lines and both treatment groups, following different dynamics at 1 and 5 DPT, aligns with the labile iron-driven reliance on GPX4. Critically, these cells are not spontaneously dying from ferroptosis (Figure S6, DMSO-treated conditions), reinforcing the fact that surviving cell populations are managing their labile iron burden by relying on GPX4 and its regeneration by GSH. Ferroptosis has been reported as a form of immunogenic cell death in some settings that, upon the loss of membrane integrity, may increase immunogenicity and activate initiate immunity [44]. We evaluated common molecular markers of immunogenicity, including ATP and HMGB1 release, as well as M-CSF and IL-2, in cells at 10 DPT co-cultured with PBMCs, both before and after ferroptosis (Supplementary Figure S7). Ferroptotic cell death at 10 DPT did not release commonly recognized immunogenic molecules, although more research is warranted to identify additional immunogenic aspects of ferroptosis.

## 3. Discussion

**Figure 5:**
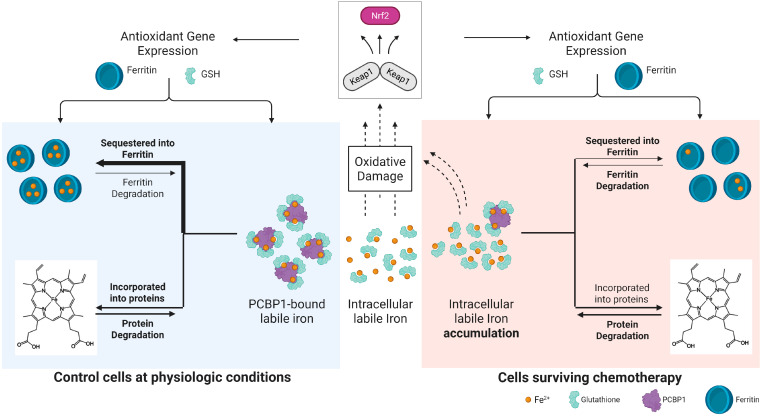
Mismanagement of labile iron in cells surviving chemotherapy. (Left, in blue) when labile iron accumulates intracellularly, it causes oxidative damage that leads to NRF2 activation and the upregulation of antioxidant and iron-handling proteins, such as GSH and ferritin. Physiologically, this cellular response leads to increased ferritin expression and PCBP1-mediated transport of Fe-GSH complexes to the ferritin nanocages for iron inactivation and sequestration. In this study, we show that cells surviving chemotherapy have active NRF2 signaling and competent GSH and ferritin enrichment, but labile iron is not being sequestered, likely due to the relative lack of the PCBP1 chaperone (right, in red). Created in BioRender. Amend, S. (2025) https://BioRender.com/3c6fbfr (accessed on 24 April 2025).

In this study, we sought to leverage ferroptosis as a proliferation- and apoptosis-agnostic mechanism for the elimination of resistant cancer. We utilized two different cell lines and chemotherapies and identified a convergent phenotype that arises over time, such that cells that have survived 10 days post-chemotherapy, regardless of cell line or chemotherapy, display similar morphology and transcriptomic signatures. Surviving cells at 10 DPT have high amounts of labile iron and display characteristics of iron toxicity, where NRF2 activation leads to GSH pathway enrichment and high ferritin expression. However, ferritin is not alleviating labile iron levels, nor can labile iron levels be explained by ferritinophagy. Instead, we find that a relative loss of PCBP1 expression is the most likely cause of labile iron accumulation (Figure 5). A shared labile iron burden in this convergent phenotype creates a similar reliance on GPX4 and, thus, vulnerability to ferroptosis, despite different sensitivities to ferroptosis in untreated cell lines. Our study highlights a convergent phenotype with a shared, labile iron-driven vulnerability to ferroptosis. Vulnerability to ferroptosis counterintuitively occurs along with NRF2 activation, high GSH levels, and high ferritin expression in surviving cells.

PCBP1 is the primary cellular iron chaperone and is required for iron loading into ferritin, cytosolic [2Fe-2S] clusters, and directly into enzymes [25,26]. Ten DPT cells are reminiscent of PCBP1-deficient models that have poor ferritin loading, increased labile iron pool, and lipid peroxidation [23,31]. The loss of PCBP1 in surviving cells likely explains the counterintuitive vulnerability to ferroptosis when the NRF2, GSH, ferritin axis is enriched. High levels of GSH in cells at 10 DPT are needed to both bind excess labile iron and manage redox requirements, including regeneration of the GPX4 active site, allowing cells at 10 DPT to avoid spontaneous ferroptotic cell death. However, without attenuating the source of lipid peroxidation, i.e., high labile iron, surviving cells are reliant on lipid peroxidation defenses and susceptible to GPX4 inhibition. Deficient iron trafficking from PCBP1 loss may also be impacting the function of other iron-containing enzymes in cells at 10 DPT, particularly in the mitochondria and nucleus, and contributing to the convergent transcriptomic signature. In addition to chaperoning iron, PCBP1 has separate roles in RNA processing and has been implicated for gene regulation in epithelia–-mesenchymal transition and innate immune signaling, as well as binding oxidized RNA [45,46,47]. While it is tempting to speculate that the loss of PCBP1 is related to RNA metabolism imbalances or activation of one pathway enabling chemotherapy survival, while consequently contributing to labile iron accumulation, more research is needed to tease out PCBP1′s distinct and independently vital roles [30].

The dysregulation of cellular iron homeostasis is causal or characteristic to many diseases, and ferroptosis is an increasingly appreciated contributor to many pathologies [38,48,49,50]. Labile iron accumulation in cells 10 DPT was visibly punctate compared to untreated cell lines, with much of the punctate staining colocalizing with active lysosomes. Lysosomal accumulation of iron is characteristic of chronic stress conditions, particularly neurodegenerative diseases, but whether iron sequestration in lysosomes is protective or not is unclear. On the one hand, containing iron in lysosomes prevents diffusion and labile iron’s access to other cellular compartments. However, lysosomal iron is not detoxified as it is in ferritin, and thus causes lipid peroxidation, increased lysosomal permeability, and oxidation of proteins that accumulate in insoluble aggregates [20,38,50]. In regard to ferroptosis, lysosomal iron is known to be essential for ferroptosis execution due to deferoxamine’s ability to prevent ferroptosis in most contexts [3]. Indeed, deferoxamine prevented ferroptotic cell death by GPX4 inhibition in all samples in this study (Supplementary Figure S6). Additionally, a recent study showed that lysosomal iron can trigger ferroptosis by initiating lipid peroxidation in lysosomes that then propagate to ER and other intracellular membranes [51]. Intracellular localization of labile iron remains a critical underexplored area of ferroptosis research, and lysosomal ferrous iron pools constitute a novel target for increasing sensitivity to ferroptosis in resistant cancer settings [51,52].

Ferroptosis is an emerging vulnerability in resistant cancer settings. In our study, cells surviving chemotherapy are also vulnerable to ferroptosis, but this vulnerability is driven by labile iron accumulation, partially in lysosomal compartments, and occurs in spite of active NRF2 signaling. The inability of ferritin to relieve labile iron burden is also counterintuitive. Metabolic programming in cells surviving chemotherapy remains unexplored here, including NRF2-driven rewiring of metabolic programs and the complex relationships between metabolite abundance, autophagy, and lipid composition [3,53,54]. The labile iron-driven phenotype in this study reinforces the highly flexible mechanisms involved in ferroptosis and the importance of timing when considering therapeutic strategies [9]. Iron homeostasis dynamics in cancer, intracellular localization of reactive iron, and the impact of PCBP1 on intracellular iron handling, are important areas of further research to enable the translation of ferroptosis therapeutic strategies.

## 4. Materials and Methods

### 4.1. Cell Culture and Generation of Surviving Cells

PC3 cells (CRL-1435) and MDA-MB-231 cells (HTB-26) were obtained from ATCC. PC3 cells were cultured in RPMI (Gibco #11875119, Billings, MT, USA) with 10% fetal bovine serum (FBS, Avantor #97068-085, Radnor, PA, USA) and 1% penicillin–streptomycin. MDA-MB-231 cells were cultured in DMEM (Gibco #11995073) with 10% FBS and 1% penicillin–streptomycin. Surviving cells were generated by treating with IC50 cisplatin or docetaxel for 72 h, then chemotherapy was removed and cells maintained in fresh media with routine passaging. PC3 chemotherapy treatment was 6 µM or 5 nM docetaxel. MDA-MB-231 treatment was 12 µM cisplatin or 5 nM docetaxel.

### 4.2. Drug Treatments

Drugs used in this study are Bafilomycin (SelleckChem #S1413, 1 µM); deferoxamine mesylate (SelleckChem #S5742, Houston, TX, USA, 50 µM, DFO); Ferrostatin-1 (SelleckChem #S7243, 1 µM, Fer-1); ML210 (SelleckChem #S0788, 1 µM).

### 4.3. Labile Iron Live Cell Imaging

Cells were lifted using TypLE Express (Gibco #12604039) (Thermo Fischer, Waltham, MA, USA) and re-plated on to 35 mm IbiTreat µ-Dishes (Ibidi, Inc., Fitchburg, WI, USA). Untreated cell lines were seeded the day prior to imaging. For 1 DPT, cells were collected and re-plated at the end of chemotherapy treatment. For 5 DPT cells, cells were collected and re-plated at 3 DPT. For 10 DPT cells, cells were collected and re-plated at 7 DPT. On timepoint day, dishes were washed once with HBSS (Gibco #14025-092) and then incubated with FerroOrange (Millipore Sigma #SCT210, Rockville, MD, USA, 1 µM); ER-Tracker Green (BODIPY™ FL Glibenclamide) (Invitrogen #E34251, Waltham, MA, USA, 1 µM); and Hoechst 33342 (Invitrogen, Thermo Fischer, Waltham, MA, USA #I35101B, 1 µM) in HBSS for 30 min at 37 °C. Dishes were then washed twice with HBSS and left in HBSS for imaging. Imaging was performed one dish at a time, with no more than 30 min elapsing before completion of imaging. Images were acquired using a Zeiss Observer Z1 microscope/ZEN pro 2.0 software (Carl Zeiss Microscopy, White Plains, NY, USA) with a Axiocam MRm camera (Zeiss, White Plains, NY, USA) and X-Cite Xylis LED Fluorescence Light Source (Excelitas Technologies, Boulder, CO, USA). Images were background-subtracted using Rolling Ball background subtraction, manual ROIs were drawn per cell using phase and Hoechst imaging, then integrated density was measured per cell using Fiji [55]. For lysosomal colocalization, FerroOrange staining was the same as described, plus LysoTracker Deep Red (Invitrogen #L12492, 50 nM) was spiked into the final 15 min of staining time period. Image acquisition was performed as described, using higher magnification objective. Colocalization analysis was performed using Fiji (version 2.9.0).

### 4.4. Labile Iron Assay from Lysates

On timepoint day, cells were lifted using TypLE Express, washed once in HBSS, counted with hemacytometer and then snap frozen. An assay was run no more than 7 days after snap freezing. For the assay, cell pellets were lysed using Mammalian Cell Lysis Buffer 5X (Abcam, Waltham, MA, USA #ab179835) with Halt Protease and Phosphatase Inhibitor Cocktail (Thermo Scientific, MA, USA #78440) added. Iron Assay Kit (colorimetric) (Abcam #ab83366) was then run according to the manufacturer’s protocol.

### 4.5. Western Blot

On timepoint day, cells were lifted using Cell Dissociation Solution Non-enzymatic 1X (Millipore Sigma, St. Louis, MO, USA #C5914), washed once in PBS and then snap frozen. Cell pellets were lysed no more than 7 days after snap freezing. Cell pellets were lysed using RIPA buffer with Halt Protease and Phosphatase Inhibitor Cocktail (Thermo Scientific, MA, USA #78440). Protein concentration was determined by bca assay. Western blot samples were prepared using Laemmli Sample Buffer (Bio-Rad, Hercules, CA, USA) supplemented with β-mercaptoethanol (Bio-Rad, CA, USA). The same total protein amounts were loaded into each lane of 4–20% Mini-PROTEAN TGX Stain-Free Protein Gels (Bio-Rad, CA, USA). Completed gels were transferred onto 0.2 µm nitrocellulose membrane. Membranes were blocked using Casein Blocking Buffer (Millipore Sigma, MO, USA #B6429) and then incubated overnight in primary antibodies diluted in blocking buffer. Membranes were washed using Tris-buffered saline with 0.1% Tween 20 after incubation in both primary and secondary antibodies. Membranes were imaged using Li-COR Odyssey system. Blots were quantified in Fiji with each target protein densitometry normalized to respective actin loading control densitometry.

Antibodies used in this study were B-actin (Sigma-Aldrich #A5441, RRID:AB_476744, 1:5000, Saint Louis, MO, USA); ferritin (Abcam #ab75973, RRID:AB_1310222, 1:800); transferrin receptor (Abcam #ab269513, RRID:AB_3351672, 1:3000); NRF2 (Abcam #ab62352, RRID:AB_944418, 1:800); PCBP1 (Abcam #ab168377, RRID: AB_3665910, 1:1000); GPX4 (Abcam #ab125066, RRID:AB_10973901, 1:1000); LC3B (Cell Signaling Technology #3868, RRID:AB_2137707, 1:1000); p62 (Cell Signaling Technology #39749, RRID:AB_2799160, 1:1000); IRDye 680RD Goat anti-Mouse IgG Secondary Antibody (LICOR Biosciences #926-68070, Lincoln, NE, USA, RRID:AB_10956588, 1:20,000); IRDye 800cw Goat Anti-Rabbit IgG Secondary Antibody (LI-COR Biosciences, NE, USA #926-32211, RRID:AB_621843, 1:12,000).

### 4.6. GSH/GSSG Assay

Cells were plated into 96 well plates prior to assay being run (as described in the ‘Labile iron live cell imaging’ section). GSH/GSSG ratio was determined using GSH/GSSG-Glo Assay kit (Promega, Madison, WI, USA #V6611) according to the manufacturer’s protocol. Both untreated cancer cell lines were included in every assay plate as a control. Any wells that were >80% confluent were not assayed. All reagents were made prior to washing and on plate lysis, as described in the protocol, to ensure rapid assay completion and as accurate a determination of GSH/GSSG ratio as possible.

### 4.7. C11 BODIPY Analysis

Cells were seeded into 24 well plates (as described in the ‘Labile iron live cell imaging’ section). C11 BODIPY 581/591 (Invitrogen# D3861, 1 µM) dissolved in HBSS was added to each sample. Cells were incubated for 30 min at 37 °C, washed once in HBSS, then imaged in HBSS. Images were acquired using an EVOS M7000 imaging system. Images were quantified in Fiji by manually drawing ROIs per cell using phase and Hoechst images. Red and green fluorescence values were background-corrected by quantifying the red or green fluorescence in cell-free areas from at least four cell free ROIs and subtracting from per-cell values to determine the final fluorescence values. C11 BODIPY ratio is green fluorescence (oxidized)/red + green fluorescence total).

### 4.8. Ferroptosis Cell Death Assay

Cells were seeded into 6 well plates (as described in the ‘Labile iron live cell imaging’ section). Cells were treated with DMSO, ML210, ML210 and Fer-1, or ML210 and DFO in DMEM supplemented with Incucyte Cytotox Green Dye (Sartorius, Bohemia, NY, USA #4633). Live cell timelapse microscopy was performed using an Incucyte SX5 (Sartorius) with imaging every hour for 10 h. Movies were assembled in Fiji and manually counted to determine cell loss (detachment from apoptosis) and cell death by ferroptosis (adherent, but positive for Cytotox Green, at 10 h). Every plate included one well for each condition, and 100~150 cells were counted per well across at least two fields of view. Ferroptosis was confirmed by Fer-1 and DFO fully preventing ferroptotic cell death.

### 4.9. Release of Immunomodulatory Markers During Ferroptosis

Immunomodulatory molecule detection experiments were performed at Cellomatics Biosciences, 10 Colwick Quays Business Park, Private Road No.2, Colwick, Nottingham NG4 2JY, United Kingdom.

Cells were seeded into 96 well plates as described. On assay day, cells were treated with DMSO or ML210 for 6 h prior to co-culture with PBMCs. Extracellular ATP release was performed using the RealTime-Glo Extracellular ATP Assay (Promega #GA5010) according to the manufacturer’s protocol. HMGB1, M-CSF, and IL-2 were detected via ELISA (ThermoFisher #EEL047) and Luminex Multiplex Assay (R&D Systems, MN, USA #FCSTM18B-10).

PBMCs were isolated as follows. Three healthy blood donors were recruited, and fresh whole blood was collected on the day of co-culture for each timepoint into BD vacutainer tubes coated with EDTA (Becton Dickinson, UK #367525). PBS was added to the whole blood collected in EDTA-coated vacutainer tubes at equal volumes, and the tubes were mixed gently. Approximately 15 mL of Lymphoprep (StemCell Technologies, BC, Canada #7801) was added into SepMate tubes (StemCell Technologies, MA, USA #85450), which were kept vertical in a tube rack. The diluted blood sample was then gently pipetted down the sides of the tubes. The tubes were centrifuged at 1200× g for 12 min at room temperature. The PBMCs above the plastic insert of the SepMate tube were transferred into a 50 mL tube. PBMCs were washed with 10 mL PBS. The tubes were centrifuged at 1200× *g* for 12 min at room temperature. The PBMC pellets were resuspended in 1× RBC lysis buffer and incubated at room temperature for 10 min before being centrifuged at 600× *g* for 7 min. The PBMC pellets from each donor were then resuspended in pre-warmed complete media (the same media used for cells) and counted based on trypan blue dye exclusion. Equal PBMC numbers were pooled from each donor for the co-cultures.

### 4.10. Statistical Analysis

All statistical analyses were performed in GraphPad Prism version 9.1 (GraphPad, LLC., MA, USA.). An α = 0.05 (confidence level 95%) was the criterion considered to determine statistical significance for all tests. No significance (ns), *, **, ***, and **** represent *p* values of ≥0.05, <0.05, <0.01, <0.001, and <0.0001, respectively.

## Data Availability

The original contributions presented in this study are included in the article/Supplementary Materials. Further inquiries can be directed to the corresponding authors.

## Supplementary Materials

The following supporting information can be downloaded at: https://www.mdpi.com/article/10.3390/ijms26094193/s1.
