# Supplementary material for: Dysregulation of Labile Iron Predisposes Chemotherapy Resistant Cancer Cells to Ferroptosis
Source: Int J Mol Sci. 2025 Apr 28;26(9):4193. doi: 10.3390/ijms26094193 (PMC12072162; doi:10.3390/ijms26094193)
Supplement: Supplementary file 1 [file ijms-26-04193-s001.zip › ijms-3578290-supplementary.pdf]

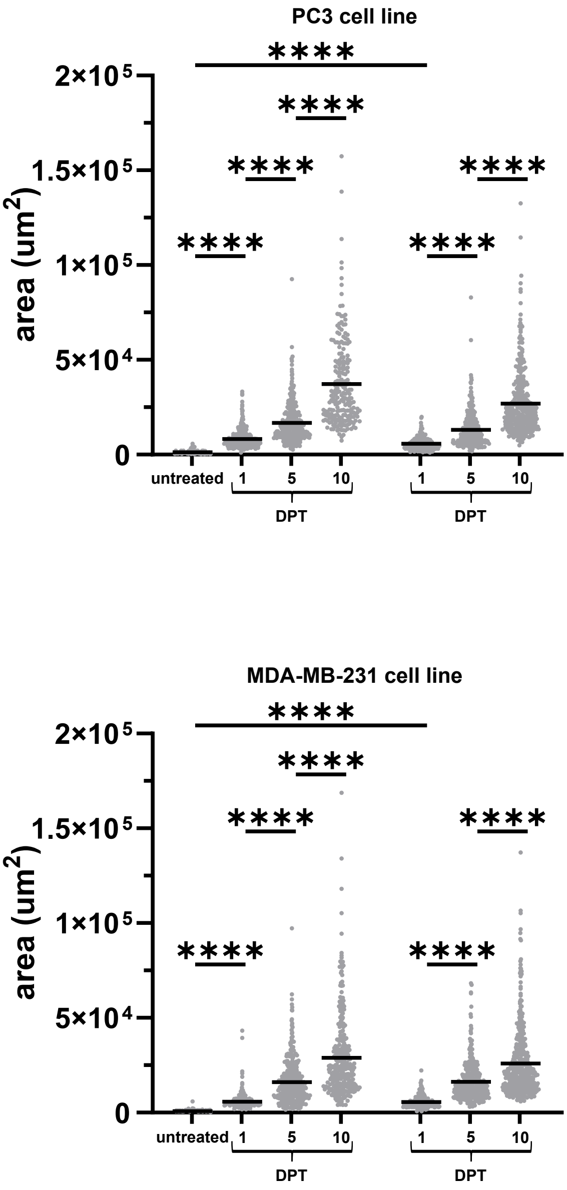

Supplementary Figure S1 Cells surviving chemotherapy increase in size over time.
Area of PC3 and MDA-MB-231 cell lines before and after cisplatin or docetaxel as determined by live cell phase contrast imaging.

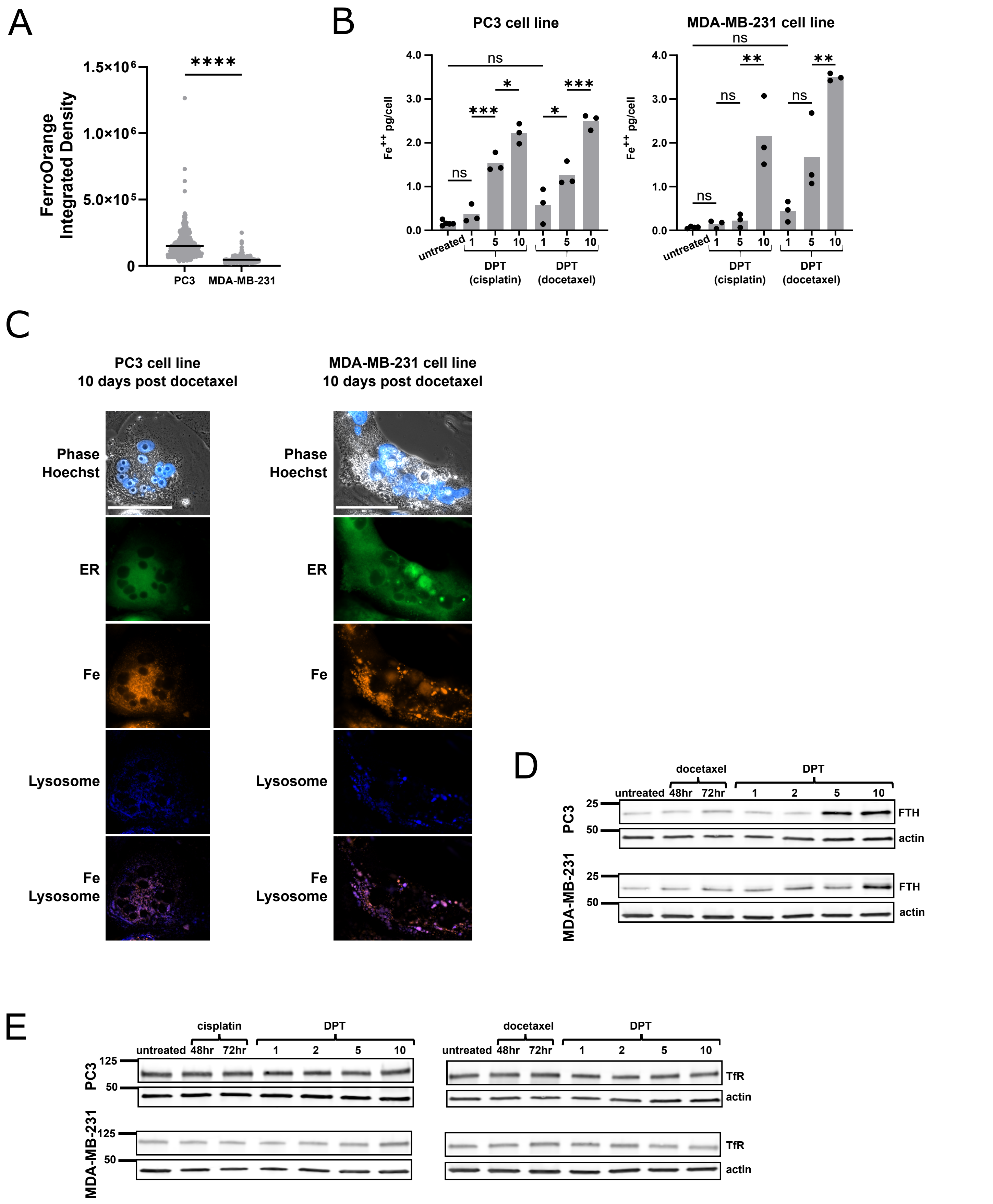

Supplementary Figure S2. Labile iron is dramatically elevated by 10 days recovery from chemotherapy. A. Quantification of FerroOrange integrated density in untreated cell lines from Fig. 3A. Data displays individual cells (dot) and mean (bar) from three biological replicates **B.** Labile iron assay from cell lysates of PC3 and MDA-MB-231 cells before and after chemotherapy. **C.** Live cell images with FerroOrange (Fe), ERTracker Green (ER), and LysoTracker (Lysosome) of PC3 and MDA-MB-231 cells 10 days after cisplatin. Quantification in Figure 3D. **D.** Ferritin expression in PC3 and MDA-MB-231 cells before and after docetaxel. **E.** Transferrin Receptor expression in PC3 and MDA-MB-231 cells before and after cisplatin or docetaxel.

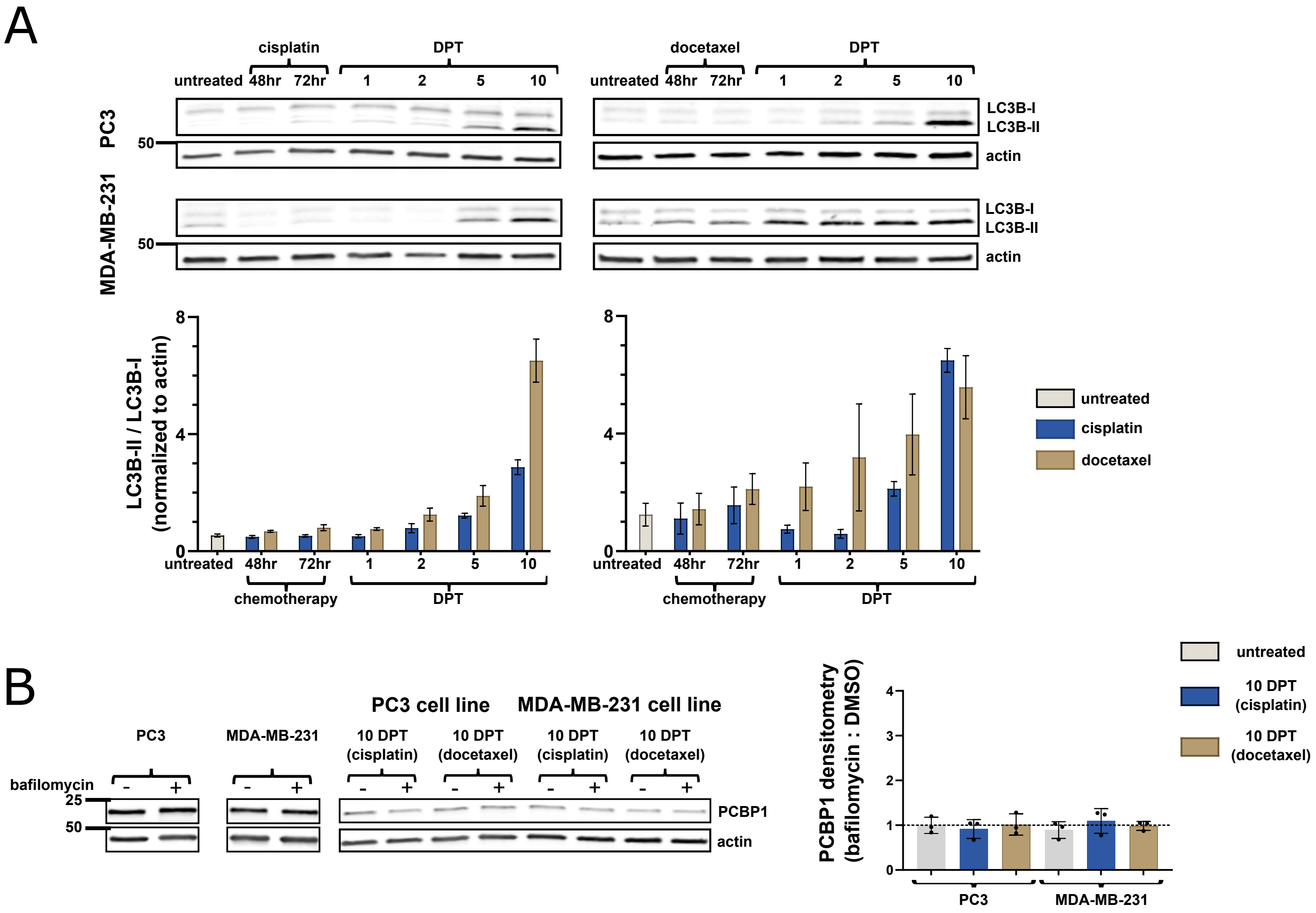

Supplementary Figure S3. Enriched LC3B protein expression in cells surviving chemotherapy. A. Western blot for LC3B in PC3 or MDA-MB-231 cells surviving cisplatin of docetaxel. Quantification is LC3B-II / LC3B-I ratio. **B.** Western blot for PCBP1 expression following 1 uM bafilomycin or DMSO treatment for 6 hrs. Quantification shows bafilomycin : DMSO treated densitometry ratio. Actin bands same as in Fig. 2E.

A

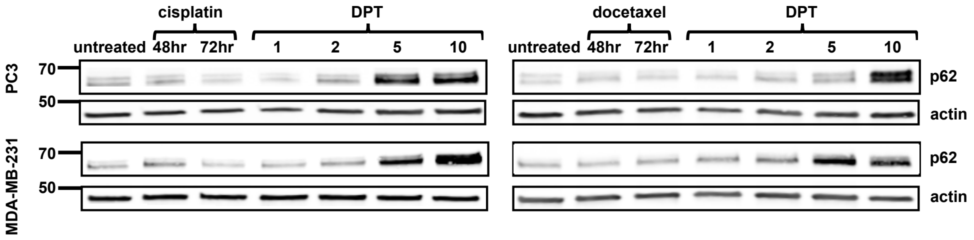

Supplementary Figure S4. Surviving cells 10 days post chemotherapy have enriched p62. A. Western blot for p62 expression in PC3 and MDA-MB-231 cells following cisplatin or docetaxel treatment.

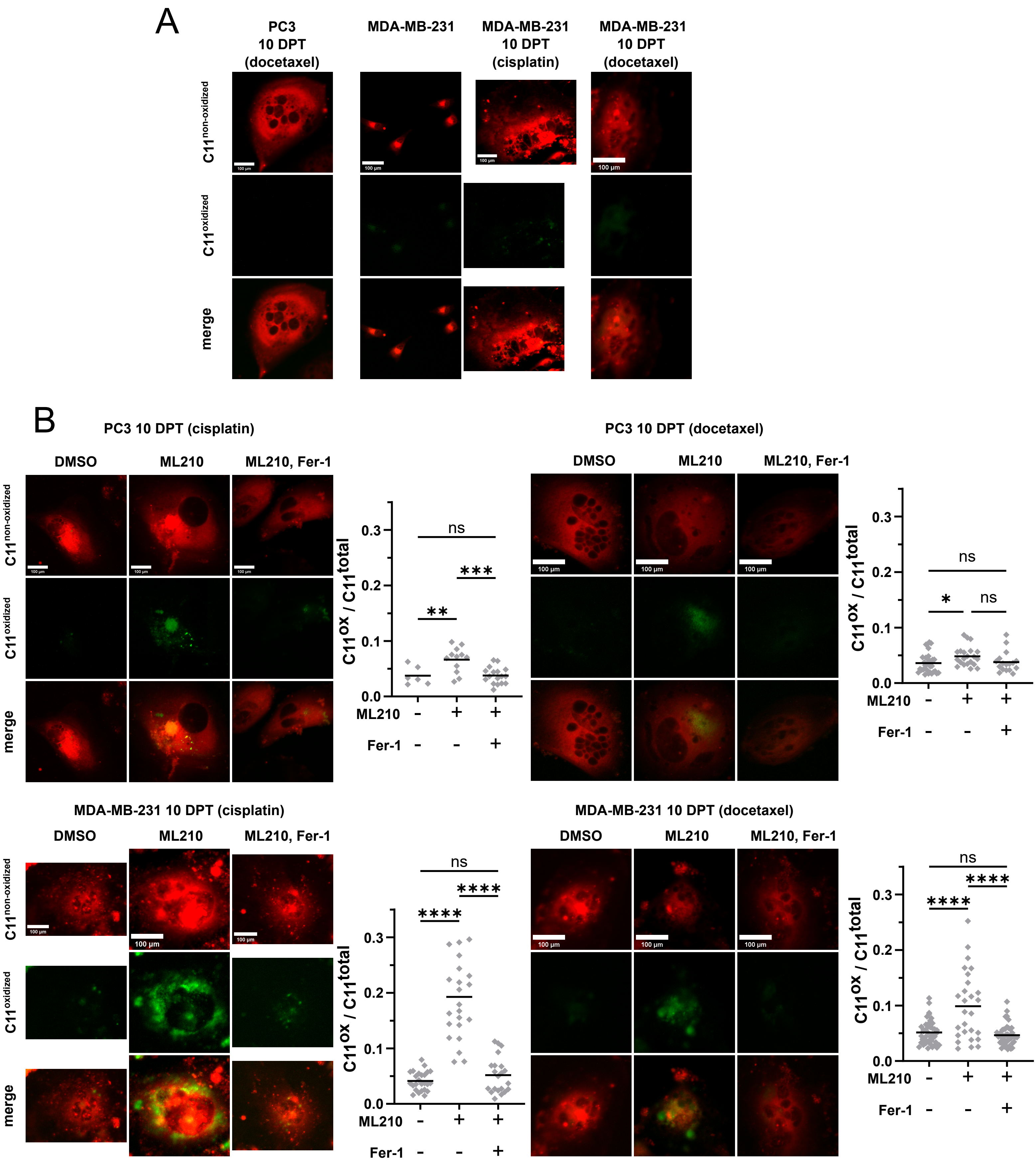

Supplementary Figure S5 GPX4 inhibition leads to a build up of oxidized lipids. A. C11 Bodipy imaging in PC3 cells 10 days post docetaxel, and MDA-MB-231 cells untreated and 10 days post cisplatin or docetaxel. Quantification in 6A. **B.** C11 Bodipy imaging and quantification for all 10 DPT surviving cell contexts treated with DMSO, ML210, or ML210 and Ferrostatin-1 (Fer-1) treatment for 6 hours. Quantification is ratio of oxidized (green) to total (green + red) C11 Bodiy fluorescence.

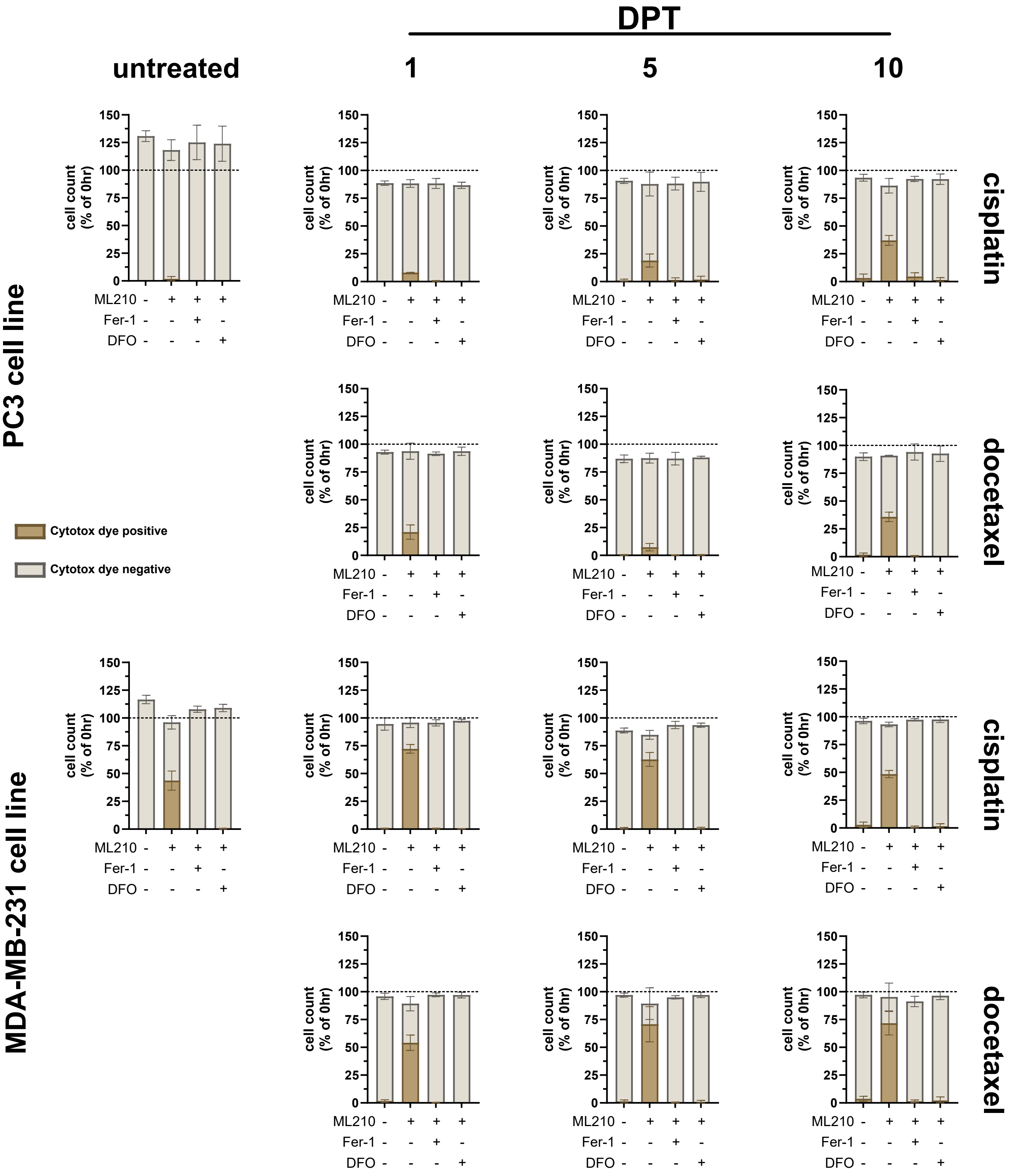

Supplementary Figure S6 Ferrostatin-1 and Deferoxamine prevent ferroptotic cell death from GPX4 inhibition. All untreated cell lines and timepoints of surviving cells following chemotherapy treated with, from left to right, DMSO (0.1%); ML210 (1uM); ML210 and Fer-1 (1uM); ML210 and DFO (50uM). Percent of total cell count is change in cell number over 10 hours from cell detachment (apoptosis) or cell proliferation. Cytotox Green dye positive cells are still adherent at end of timelapse (10 hours). PC3 cells 10 DPT (cisplatin) are repeated from Figure 6D.

Fer-1 = Ferrostatin-1

DFO = Deferoxamine

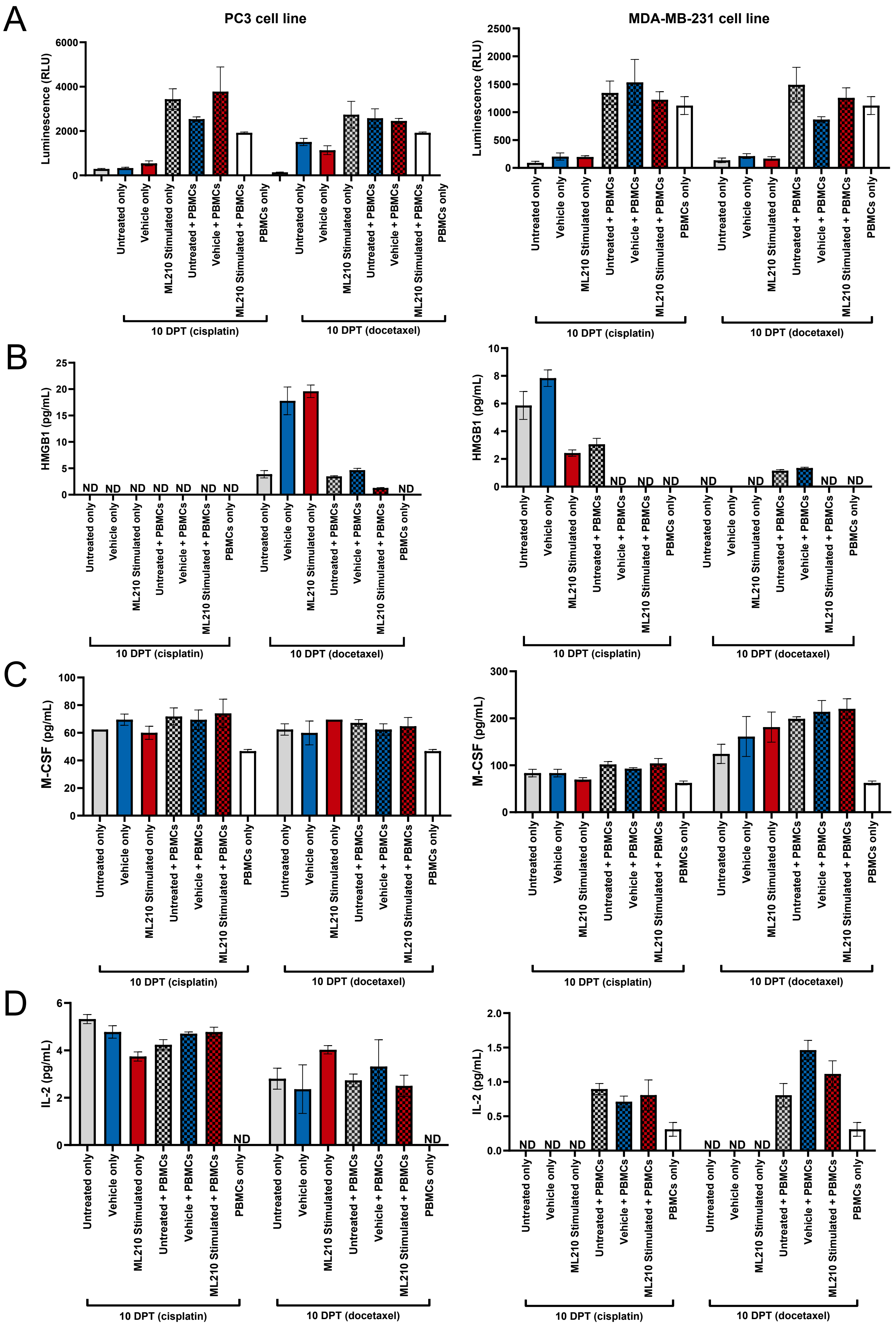
A
PC3 cell line
MDA-MB-231 cell line
Luminescence (RLU)
B
HMGB1 (pg/mL)
C
M-CSF (pg/mL)
D
IL-2 (pg/mL)
ND
Untreated only
Vehicle only
ML210 Stimulated only
Untreated + PBMCs
Vehicle + PBMCs
ML210 Stimulated + PBMCs
PBMCs only
10 DPT (cisplatin)
10 DPT (docetaxel)

Supplementary Figure S7 Ferroptotic cell death does not release immunogenic molcules. A. PC3 or MDA-MB-231 surviving cells 10 days post chemotherapy and treated with ML210 were assessed for ATP (**A**), HMGB1 (**B**), M-CSF (**C**), and IL-2 (**D**) in presence or absence of human PBMCs.
